# Management of the malnourished patient: it’s now time to revise the guidelines

**DOI:** 10.1186/s40337-022-00539-4

**Published:** 2022-04-19

**Authors:** Michael R. Kohn, Neville H. Golden

**Affiliations:** 1 AYA Medical Services WSLHD, AYA Medicine, Westmead Hospital, CRASH Centre for Research Into Adolescent’S Health, Faculty of Medicine and Dentistry, Sydney University, Sydney, Australia; 2 Division of Adolescent Medicine, The Marron and Mary Elizabeth Kendrick Professor of Pediatrics, Stanford University School of Medicine, 750 Welch Road, Suite 210, Palo Alto, CA 94304 USA

Restrictive eating disorders such as anorexia nervosa and atypical anorexia nervosa typically result in development of protein energy malnutrition. In severe protein energy malnutrition, alterations occur in all organ function, metabolism, and endocrine systems. Electrolyte levels must be closely monitored, particularly after initiating refeeding to avoid the refeeding syndrome, a rare but potentially deadly complication of refeeding the malnourished patient [1–5]. Anorexia nervosa is estimated to have the highest mortality of any psychological illness [6]. Approximately half of the mortality of anorexia nervosa occurs around the time of diagnosis, and those with severe malnutrition are most vulnerable.

Weight and BMI are the most commonly used terms to categorise the severity of malnutrition. In recent years, our understanding of severe malnutrition has evolved with the recognition that individuals with eating disorders can demonstrate the physical and psychological effects of malnutrition even at normal or above normal weights. A new diagnostic category in DSM-5 “Atypical Anorexia Nervosa” describes those individuals who have lost a significant amount of weight but whose weight is in the normal or above normal range [7]. Weight suppression, the difference between highest weight and current weight, is a predictor of medical outcome in patients with restrictive eating disorders [8, 9]. In adults, a BMI < 15 kg/m^2^ is recognised as conferring severe malnutrition. In adolescents, a BMI < 70% median BMI (corresponding approximately to a BMI < 5th percentile) indicates severe malnutrition. The Society for Adolescent Health and Medicine (SAHM) has expanded the definition of severe malnutrition in adolescents and young adults to include not only a low BMI, but also includes those who have lost > 20% of body mass over a period of one year or > 10% body mass over six months, in recognition of the effect of weight suppression on severity of medical complications [10]. This special edition of the journal addresses a number of important issues in the medical management of the malnourished patient with an eating disorder. Herein investigators provide data that challenge the existing consensus-based guidelines for refeeding.

Gibson et al. describe the medical findings of 281 severely malnourished adult patients with extreme anorexia nervosa (BMI < 15 kg/m^2^, < 65% IBW) hospitalized on their medical stabilization unit [11]. On admission, 56% had bradycardia, 45% elevated liver function tests, 64% leukopenia and 20% thrombocytopenia. With refeeding, 38% developed hypoglycemia, 35% developed hypophosphatemia, and 33% developed edema. Almost 90% of the patients had low bone mineral density. Markedly elevated liver function tests predicted hypoglycemia and low BMI predicted refeeding hypophosphatemia.

In an echocardiographic study of 124 severely malnourished adult patients with anorexia nervosa, Hanachi et al. [12] found that subclinical myocardial impairment was common. In their study, 27% of participants had pericardial effusions and 15% had evidence of left ventricular systolic dysfunction, which was associated with elevated transaminases and a diagnosis of anorexia nervosa – binge eating purging type.

Taken together, these two studies demonstrate the high rates of medical complications in severe anorexia nervosa.

The use of consensus-based guidelines has continued to inform assessment and refeeding practices in anorexia nervosa. This consensus-based approach [13–15], has promoted a “start low and go slow” approach to refeeding, which is not reported to have resulted in avoidance of refeeding complications. Several authors have commented that use of these guidelines results in “under feeding syndrome” and extended inpatient length of stay [16–28]. In an adolescent population, Schlapfer et al. [29] retrospectively studied 291 patients, 154 of whom were treated with high calorie refeeding (> 1500 kcals/d) and 137 low calorie refeeding (< 1500 kcals/d), adjusting for severe malnutrition using the updated SAHM guidelines. They demonstrated that high calorie refeeding was associated with shorter length of stay and no increased rates of electrolyte disturbances, cardiac complications, or ICU admissions, echoing the findings of others. Their results add to the growing body of literature challenging the conservative “start low and go slow” approach to refeeding recommended by many organizations [13–15].

Parker et al. [30] explore preconceptions relating to thiamine levels in adolescents with anorexia nervosa and mild to moderate malnutrition. Whilst the decision to supplement with thiamine is a relatively low cost and minor consideration when commencing refeeding, this study demonstrates that on 60 consecutive admissions to an adolescent and young adult inpatient eating disorders unit, thiamine deficiency was uncommon and adequate serum thiamine levels were rapidly achieved using standard enteral (oral and/or nasogastric) refeeding. In their series of 60 patients, no patient developed thiamine deficiency after refeeding was initiated, again challenging the NICE recommendation to supplement all patients with thiamine empirically before refeeding is started.

In this special edition, two papers examine the effect of macronutrient content on refeeding outcomes, an important yet understudied topic. Theoretically, a high carbohydrate load has been postulated to increase the risk of hypophosphatemia by stimulating an insulin surge and driving phosphorus and other electrolytes intracellularly [31]. The presumed adverse outcome of high calorie refeeding with carbohydrate as the main energy source is challenged in the article by Draffin et al. [32]. In this pilot study, 23 adolescent patients with anorexia nervosa were randomised to receive an isocaloric oral feeding regime of either high carbohydrate (50–60% energy content) or low carbohydrate (< 40% energy content). Similar rates of weight gain were seen for both groups across the admission without hypophosphatemia occurring in either group. Initial weight gain was greatest in the high carbohydrate group, though the authors reflect this may be from greater fluid shifts at the beginning of refeeding in the high carbohydrate group.

Refeeding outcomes with variation in macronutrient content were also assessed by Parker et al. [33]. Their study compared the efficacy and safety of an iso-caloric lower carbohydrate/high fat enteral formula (28% carbohydrate, 56% fat) against a standard enteral formula (54% carbohydrate, 29% fat) in 24 patients (aged 15–25 years) hospitalised with anorexia nervosa. In this double blind randomised controlled trial there was no significant difference in weight gain between the two groups over the first two weeks of refeeding, but a significantly lower rate of hypophosphatemia was found in patients who received the enteral feed with lower carbohydrate/high fat compared to standard formula. Serum phosphorus levels decreased in both groups, however it decreased to a larger extent in the standard feed group compared to the lower carbohydrate/high fat feed group.

Both the Draffin and the Parker papers had small sample sizes and one studied oral refeeding while the other addressed enteral refeeding. More research of the optimal method and macronutrient content for refeeding is needed to better inform clinical practice.

Taken together, the articles in this special edition of the journal demonstrate that severe malnutrition in restrictive eating disorders is associated with significant medical complications but also illustrates the importance of challenging existing consensus- based treatment recommendations regarding nutritional rehabilitation. The time has now come to replace existing refeeding guidelines with recommendations that are evidence-based.
